# Mortality and associated influencing factors among oral cancer patients in western China: A retrospective cohort study from 2016 to 2021

**DOI:** 10.1097/MD.0000000000035485

**Published:** 2023-10-13

**Authors:** Hua Li, Qiyuan Lan, Tianhua Jiang, Yuting Wu, Yaxi Wang, Wei Lu, Nuo Zhou, Xuanping Huang

**Affiliations:** a Department of Oral and Maxillofacial Surgery, College of Stomatology, Hospital of Stomatology, Guangxi Medical University, Nanning, China; b Guangxi Key Laboratory of Oral and Maxillofacial Rehabilitation and Reconstruction, Nanning, China; c Guangxi Clinical Research Center for Craniofacial Deformity, Nanning, China; d School of Public Health, Guangxi Medical University, Nanning, China.

**Keywords:** mortality, oral cancer, survival analysis, survival rate

## Abstract

Few studies have examined oral cancer-related mortality in Guangxi. This study aimed to explore the incidence and characteristics of oral cancer and to identify the risk factors for oral cancer-related mortality. The study was conducted to provide a reference for clinical treatment and to improve the survival rate of patients with oral cancer. A total of 271 patients with oral cancer who were treated in the Stomatology Hospital of Guangxi Medical University from 2016 to 2017 were selected as the research subjects. The follow-up lasted until the middle of 2021. The survival rate and mean survival time of 271 patients were calculated by the Kaplan–Meier method. Cox proportional hazard models and stratified analysis were used to explore the related factors that affect the mortality of patients. Nomogram plots were used to visualize the relationships among multiple variables. Among 271 patients with oral cancer, the 2-year and 5-year overall survival rates were 83.8% and 68.5% respectively. The results of multivariate analysis showed that, age, pathological type, surgery and readmission were significant factors affecting survival. When the above factors were incorporated into nomogram plots and stratified analysis, the results showed that the risk of death after treatment in patients with oral cancer aged > 55 years was 1.693 times higher than that in patients aged ≤ 55 years (HR, hazard ratio [HR] = 1.795, 95% confidence intervals [CI] = 1.073, 3.004). The risk of death after surgical treatment was 0.606 times higher than that without surgical treatment (HR = 0.590, 95% CI = 0.367, 0.948). Patients who were readmitted had a 2.340-fold increased risk of death compared with patients who were not readmitted (HR = 2.340, 95% CI = 1.267,4.321). Older age, surgery, and readmission were risk factors for mortality among patients with oral cancer. The median survival time of 271 patients with oral cancer was 52.0 months. Patients under the age of 55 years old and those who choose surgical treatment tend to have a better prognosis and a longer survival. Oral cancer-related mortality is affected by age, treatment mode, readmission, and other factors. All of these factors are worthy of clinical attention for their prevention and control.

## 1. Introduction

Oral cancer is one of the most common malignant tumors; it usually occurs in the oral and maxillofacial regions, such as the lips, gums, cheeks, tongue, and other parts of the oral cavity. The most common type of oral cancer is squamous cell carcinoma.^[1]^ Oral cancer is the sixth most common malignancy. In recent years, the incidence of oral cancer has increased. According to the 2020 GLOBOCAN statistics, there are more than 377,000 new cases of lip and oral cancer worldwide, and there have been more than 177,000 oral cancer-related deaths.^[2]^ The region with the highest incidence of oral cancer is Melanesia, followed by Southeast Asia, such as India and Pakistan.^[3]^ In China, according to the latest statistics from the National Cancer Center, the number of new cases of lip and oral cancer was 52,200 in 2016. Among all the cases, 36,200 were male, 16,100 were female, and 25,800 people died.^[4]^ The incidence rate of oral cancer in China is lower than the global rate, but there are large numbers of people with bad lifestyle habits, such as chewing betel nuts. Due to the rapid aging of the population, oral cancer will lead to long-term disease and economic burden for patients and their families.

The prognosis of oral cancer is related to the local economy and medical level. The disease burden of oral cancer is higher in developing countries. A study of 29 European countries found that the 5-year overall survival rate for oral cancer is approximately 50%, but after systemic therapy, the survival rate can reach 78%.^[5,6]^ In recent years, the overall 5-year survival rate of oral cancer patients in China has been approximately 64%.^[7]^ There is still a gap between our country and developed countries. According to current studies, there are several risk factors for oral cancer, such as, smoking, drinking, betel nut chewing, and HPV.^[8,9]^ Smoking and drinking are independent risk factors for oral cancer. The risk of oral cancer was found to be 2.37 times higher for smokers than for nonsmokers and 1.82 times higher for regular drinkers than for nondrinkers.^[10]^ China has the highest percentage of smokers.^[11]^ According to the statistics in 2018, there are 308 million smokers in our country. Among them, 50.5% are male and 2.1% are female.^[12]^ At the same time, the overall drinking rate among adults in China was 30.5%, (53.8% for males, and 12.2% for women).^[13]^

However, at present, few studies have examined survival among oral cancer patients in China, especially in the Guangxi region. It is difficult to conduct further research due to the difficulty of finding specific data that can systematically describe the disease situation of oral cancer. Thus, this study collected information on 271 patients with oral cancer who were treated at the Stomatology Hospital of Guangxi Medical University from 2016 to 2017. The objective of this study is to discuss the influencing factors of their mortalities and to provide a scientific basis for the prevention and control of oral cancer in the future.

## 2. Materials and methods

### 2.1. Study design and population

This observational cohort study was conducted at the Stomatology Hospital of Guangxi Medical University. We collected information on 271 patients with oral cancer who were treated at the Stomatology Hospital of Guangxi Medical University from 2016 to 2017, and the follow-up lasted until the middle of 2021. The patients included 180 males and 91 females, and their ages ranged from 15 to 92 years. We have followed up on the prognosis of the patients after treatment. This study was approved by the Human Research Ethics Committee of the College of Stomatology, Guangxi Medical University (Ethical Review No. 2022097).

### 2.2. Inclusion and exclusion criteria

The inclusion criteria were as follows: pathologically diagnosed with oral cancer; the clinical and follow-up data of the cases are complete, including age, sex, native place, nationality, occupation, tumor pathological type, and treatment status; not combined with other tumors or no medical history of tumors. Patients whose complete clinical and follow-up data were unavailable were excluded from this study.

### 2.3. Data collection

We obtained all information from the hospital electronic medical record system, including demographic characteristics, clinical and laboratory information, hospitalization time, outcomes of treatment, and date of admission to the hospital. The survival time was calculated from the earliest time of admission to the hospital. The outcome of this study was defined as death. If there was no death, the last follow-up time was used as the outcome.

### 2.4. Statistical analysis

The clinical data of 271 patients with oral cancer were collected and sorted by retrospective analysis. Patients who had the same case number but different admission times were considered as readmission cases. Analyses were performed using SPSS version 26.0, GraphPad version 8.0 and R version 4.3.0. Potential influencing factors included in the study were first analyzed by multivariate Cox regression analysis with a hazard ratio and 95% confidence intervals calculated. According to the results of Cox proportional hazard regression and by using R version 4.3.0, the nomogram model was constructed. Both internal and external verifications were performed for the nomogram plots. The survival rate and mean survival time of 271 patients with oral cancer were calculated by the Kaplan–Meier method. In the multivariate model, Cox regression was used to explore the related factors that affect the mortality of patients with oral cancer. Then, analyses were conducted after stratifying the data based on demographic variables (including sex, age, nationality, native place, and occupation), pathological type, differentiation, chronic disease, surgery and readmission. For all analyses, statistical significance was considered at *P* < .05.

## 3. Results

### 3.1. Descriptive characteristics

Among 271 patients with oral cancer, 180 (66.4%) were male, and 91 (33.6%) were female. Their ages ranged from 15 to 92 years old, with a median age of 55.79 ± 12.884 years old. As shown in Supplemental Table 1, http://links.lww.com/MD/K196, the pathological type of most oral cancer patients is squamous cell carcinoma, accounting for 92.6%. In addition, 78.6% of the patients had highly differentiated oral cancer. More than half of the patients have received surgery to treat oral cancer. Only 8.1% of patients with oral cancer had readmission records after treatment (Supplemental Table 1, http://links.lww.com/MD/K196).

### 3.2. Multivariate analysis of potential influencing factors in oral cancer patients

All potentially influencing factors were incorporated into multivariate Cox proportional hazard models. As shown in Supplemental Table 3, http://links.lww.com/MD/K198, age, pathological type, surgery, and readmission were the factors that affected the survival time of patients. Patients aged > 55 years showed poorer overall survival (1.795 [1.073, 3.004]) than those aged ≤ 55 years. Patients with adenocarcinoma and other types of oral cancer showed poorer overall survival than patients with squamous cell carcinomas (3.433 [0.798, 3.155]) (2.268 [1.002, 5.132]). Patients with surgery had better survival (0.590 [0.367, 0.948]) than patients without surgery. Patients who were readmitted after treatment showed poorer overall survival (2.340 [1.267, 4.321]) than those who were not readmitted.

### 3.3. Nomogram construction and validation

**Figure 1.**
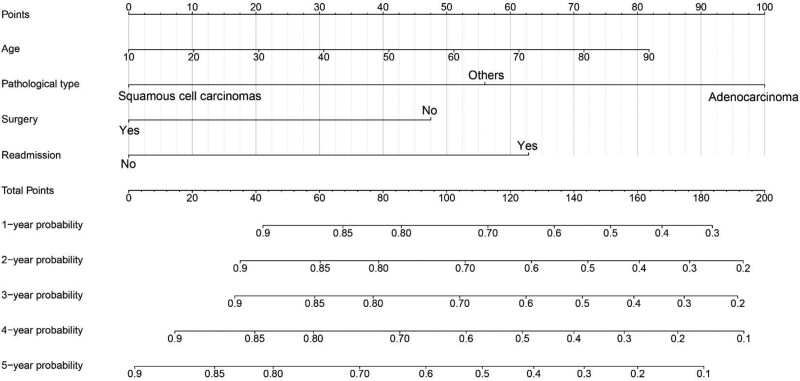
Nomogram model predicting the 2- and 5-yr survival rates of patients with oral cancer.

**Figure 2.**
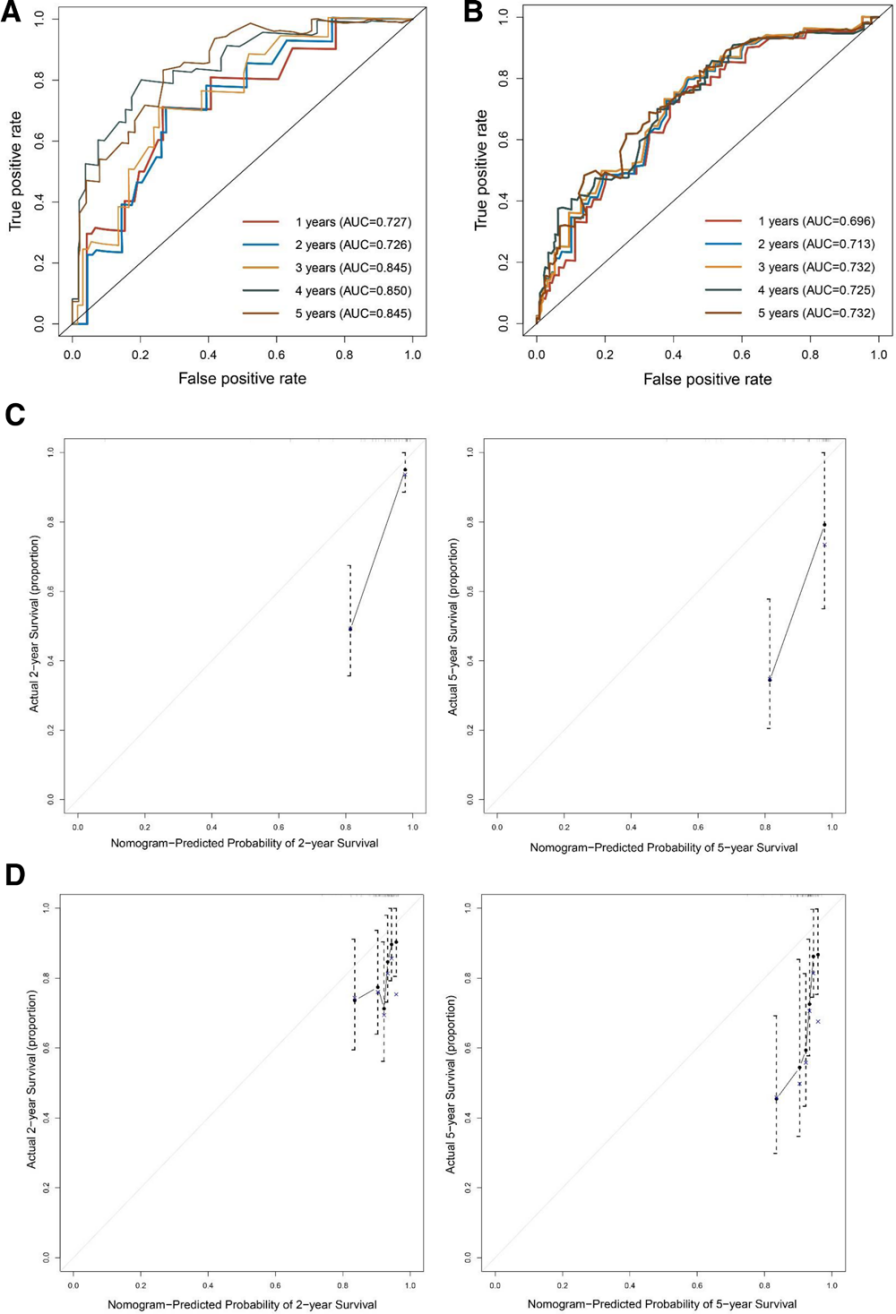
ROC curves and calibration curves of the nomogram model. (A) ROC curve of nomogram model in the training cohort. (B) ROC curve of nomogram model in the validation cohort. (C) Calibration curves of the nomogram model in the training cohort. (D) Calibration curves of the nomogram model in the validation cohort.

Based on the results of Cox proportional hazard regression, the nomogram model was constructed by using R version 4.3.0. Both internal and external verifications were performed for the nomogram plots. The nomogram plots are consistent with the multivariate analysis results, and they provide a visual representation of the scores for each variable (Fig. 1). As shown in Figure 2, the area under the curve (AUC) values at 1, 2, 3, 4, and 5 years were 0.727, 0.726, 0.845, 0.850, and 0.845, respectively, in the training cohort. And in the validation cohort, the AUC values were 0.696, 0.713, 0.732, 0.725, and 0.732, respectively. With the nomogram, the survival rate of each patient with different treatment modalities can be accurately predicted.

### 3.4. Survival rate and survival curve of potential influencing factors in oral cancer patients

**Figure 3.**
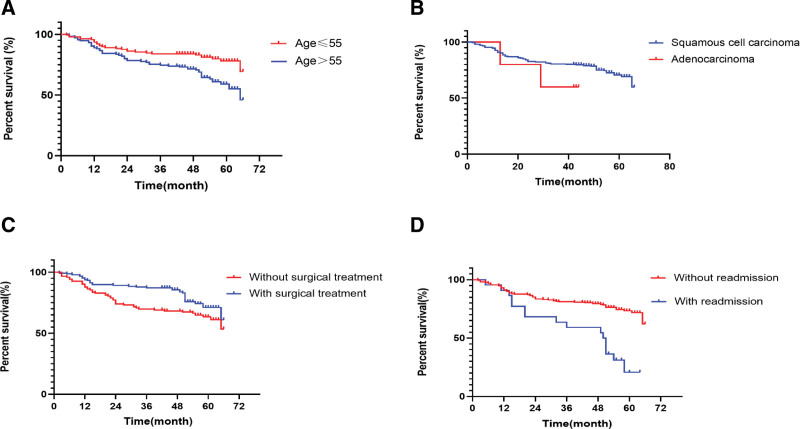
Survival curves of potential influencing factors in oral cancer patients. (A) Survival curve of patients with oral cancer by age. (B) Survival curve of patients with oral cancer by pathological type. (C) Survival curve of patients with oral cancer by surgery (with or without surgery). (D) Survival curve of patients with oral cancer by readmission (with or without readmission).

We used the Kaplan–Meier method and found that the overall 2-year survival rate of 271 oral cancer patients was 83.8%, and the 5-year survival rate was 68.5% (Supplemental Table 2, http://links.lww.com/MD/K197). Based on the survival curves, age ≤ 55, squamous cell carcinomas, with surgery and without readmission were all indicators of longer survival (Fig. 3).

### 3.5. Results of stratified analysis

As shown in Supplemental Table 4, http://links.lww.com/MD/K199, when stratifying the data by baseline characteristics that significantly differed between with-surgery and without-surgery patients, including demographic variables (sex, age, nationality, native place, and occupation), pathological type, differentiation, chronic disease and readmission, we found that the association between surgery and survival still exists (Supplemental Table 4, http://links.lww.com/MD/K199). Therefore, surgical treatment increased the survival of oral cancer patients regardless of demographic characteristics, pathological type, differentiation, chronic disease, and readmission. Surgical treatment is beneficial to the survival rate of patients with oral cancer.

## 4. Discussion

In our study, the average age of oral cancer patients was approximately 56 years old, which was similar to the findings of other studies showing that the age group with the highest incidence was 15 to 49 years old. Therefore, we use 55 years as the median age for stratification. This shows that the onset age of oral cancer is gradually decreasing.^[14]^ Previous studies have demonstrated that the incidence of oral cancer is higher in men than in women.^[3,15]^ Both the nomogram and survival curves provide strong evidence of the clinical impact that patients aged above 55 have a higher risk of mortality. Among the 271 cases in our study, the incidence of male patients was significantly higher than that of female patients. The reason may be related to the higher rates of smoking, drinking, and chewing betel nuts among males.

Moreover, squamous cell carcinoma was the most common pathological type of oral cancer in this research, while the survival rate and prognosis of squamous cell carcinoma remain low.^[16,17]^ In this multivariate analysis, the risk of death after treatment for other pathological types (e.g., malignant lymphoma and fibrosarcoma) was higher than that of squamous cell carcinoma. In addition, the characteristics of oral cancer are different in different regions. People who live in Hunan and Hainan provinces are fond of chewing betel nuts, which also increases the risk of oral cancer.^[18–20]^ The reason for the high proportion of squamous cell carcinoma in Guangxi remains unclear. This may be related to patients health habits and economic conditions.

In Guangxi, there are few investigations on the incidence of oral cancer. The occupation of these cases is almost farmers, which may indicate that the incidence of oral cancer in rural areas is higher than that in urban areas. In the multivariate analysis, we found that the risk of death after treatment in patients with other occupations was lower than that in farmers. When we collected clinical data, the specific classification was unclear. Therefore, the analysis should be carried out after the classification of occupations is clear in the future.

Currently, surgical treatment is still the main treatment for oral cancer. Surgical treatment is the mainstay of therapy for patients with oral cancer, especially in advanced stages of cancer.^[21,22]^ After surgery, some necessary adjuvant treatments, such as postoperative radiotherapy or chemoradiotherapy, should be performed according to the condition.^[23]^ However, surgical treatment is the key. In this study, patients with oral cancer had a higher survival rate after surgical treatment. According to multivariate Cox regression analysis, surgical treatment is a protective factor that contributes to the improvement of patients lives. In this study, only 8.1% of patients had referrals from our hospital, indicating that most of the patients had a good outcome after initial treatment. However, almost half of the patients in our study did not receive surgical treatment, which may be related to their own economic or physical conditions. Patients who do not receive surgical treatment have a high risk of poor prognosis and readmission. Therefore, younger patients who opt for surgical intervention are more beneficial for prognosis and quality of life.

According to the Global Burden of Disease database, we found that the incidence of oral cancer in mainland China is lower than the world average level.^[24]^ Oral cancer is considered a cancer with a poor prognosis since the 5-year survival rate is reported to be lower. Moro J.D.S. studied the case data of oral cancer patients in a hospital in southern China and found that the 5-year survival rate was 42%.^[25]^ The overall survival rate of oral cancer patients in Europe is higher. The overall 5-year survival rate for oral cancer patients in the Netherlands is 62%.^[26]^ A study carried out at the Stomatology Hospital of Peking University showed that the 5-year survival rate of oral cancer patients is 64%, which is approaching the advanced level abroad.^[27]^ In our study, the overall 5-year survival rate of 271 patients was 68.5%. It may benefit from the improvement of the overall disease awareness of the population, the propaganda and education of oral cancer health knowledge, and the improvement of oral cancer diagnosis and treatment methods. Our research has the same conclusion that the prognosis of patients with oral cancer is poor when they are over 55 years old. Senility is a significant risk factor affecting the mortality of patients, and a pervious study demonstrated that the incidence rate of oral cancer increases after 30 years of age.^[28]^ The age group with the highest incidence is 65 to 69 years old. This study also shows that patients older than 50 years old have more chronic diseases than younger patients. Although in the multivariate analysis, the presence of chronic diseases was not a statistically significant factor, in our daily life, more attention should be given to the physical health of older people.

Although the diagnosis and treatment of oral cancer has greatly improved in China, the prognosis of oral cancer patients has not improved significantly. A study showed that tumor recurrence has a great impact on the prognosis of patients and even leads to death.^[29]^ Therefore, we can timely observe whether the patient has a recurrence after surgery by regular follow-up. For patients with recurrence, early clinical treatment is helpful to prolong their survival time as much as possible and reduce their mortality.

According to this study, the survival rate of patients with oral cancer in China has gradually increased and approached the advanced level worldwide, which was encouraging. Possible reasons include increased awareness of oral healthcare, early screening and advancements in medical technology.^[30]^

This study has some limitations that should be noted. The AUC and calibration curve indicate that the model predictive ability is not satisfactory. This could potentially be due to a small sample size. In the future, it is recommended to expand the sample size to further validate the results of this study. And some clinical evaluation data are missing, it is important to enhance the capacity for data collection and organization in the future.

## 5. Conclusion

The 5-year overall survival rate of 271 patients was 68.5%. Patients younger than 55 years old who choose surgical treatment have a better prognosis and survival rate. The mortality of patients with oral cancer is affected by age, treatment method, readmission, and other factors. Therefore, early detection and treatment should be sought to improve the survival rate of oral cancer.

## Acknowledgments

We would like to express our gratitude to all staff from the Stomatology Hospital of Guangxi Medical University, for their collecting, verifying and cleaning of the data used in this study. The study was supported by Guangxi Science and Technology Base and Talents Special Project (2021AC18031), National Natural Science Foundation of China under Grant (82071098), Nanning Qingxiu District Science and Technology Plan (2021004), Guangxi Medical and Health Suitable Technology Development and Popularization Applications Project (S2021085), and Guangxi Zhuang Autonomous Region Health Health Committee Self-funded Scientific Research Project (Z-A20220744).

## Author contributions

**Data curation:** Yuting Wu.

**Investigation:** Tianhua Jiang, Yaxi Wang, Wei Lu.

**Project administration:** Nuo Zhou, Xuanping Huang.

**Writing – original draft:** Hua Li, Qiyuan Lan.

**Writing – review & editing:** Xuanping Huang.

## Supplementary Material
