# Supplementary material for: Mortality and associated influencing factors among oral cancer patients in western China: A retrospective cohort study from 2016 to 2021
Source: Medicine (Baltimore). 2023 Oct 13;102(41):e35485. doi: 10.1097/MD.0000000000035485 (PMC10578695; doi:10.1097/MD.0000000000035485)
Supplement: Supplementary file 1 [file medi-102-e35485-s001.docx]

Supplemental TABLE 1 General characteristics of 271 patients with oral cancer

|  | N | Proportion (%) |
| --- | --- | --- |
| **Gender** |  |  |
| Male | 180 | 66.4 |
| Female | 91 | 33.6 |
| **Age** |  |  |
| ≤55 | 137 | 50.6 |
| ＞55 | 134 | 49.4 |
| **Native place** |  |  |
| Guangxi | 265 | 97.8 |
| Other provinces | 6 | 2.2 |
| **Nationality** |  |  |
| Han | 164 | 60.5 |
| Zhuang | 100 | 36.9 |
| Others/Foreign | 7 | 2.6 |
| **Occupation** |  |  |
| Farmers | 131 | 48.3 |
| Others | 76 | 28.1 |
| Unemployed | 21 | 7.7 |
| Retirees | 43 | 15.9 |
| **Pathological type** |  |  |
| Squamous cell carcinomas | 251 | 92.6 |
| Adenocarcinoma | 5 | 1.9 |
| Others | 15 | 5.5 |
| **Differentiation** |  |  |
| Highly | 213 | 78.6 |
| Moderately | 28 | 10.3 |
| Poorly | 30 | 11.1 |
| **Surgery** |  |  |
| Yes | 148 | 54.6 |
| No | 123 | 45.4 |
| **Chronic diseases** |  |  |
| Yes | 69 | 25.5 |
| No | 202 | 74.5 |
| **Readmission** |  |  |
| Yes | 22 | 8.1 |
| No | 249 | 91.9 |
