# Supplementary material for: Mortality and associated influencing factors among oral cancer patients in western China: A retrospective cohort study from 2016 to 2021
Source: Medicine (Baltimore). 2023 Oct 13;102(41):e35485. doi: 10.1097/MD.0000000000035485 (PMC10578695; doi:10.1097/MD.0000000000035485)
Supplement: Supplementary file 2 [file medi-102-e35485-s002.docx]

Supplemental TABLE 3 Results of multivariate analysis of 271 patients with oral cancer

|  | Number of survival (%) | | | Number of death (%) | *HR*（95%*CI*） | *P* |
| --- | --- | --- | --- | --- | --- | --- |
| **Gender** | |  |  | |  | 0.821 |
| Male | | 130(72.2) | 50（27.8） | | 1.0 |  |
| Female | | 61(67.0） | 30（33.0） | | 1.058（0.647,1.732） |  |
| **Age** | |  |  | |  | 0.026 |
| ≤55 | | 109(79.6） | 28（20.4） | | 1.0 |  |
| ＞55 | | 82（61.2） | 52（38.8） | | 1.795（1.073,3.004） |  |
| **Native place** | |  |  | |  | 0.099 |
| Guangxi | | 188(70.9） | 77（29.1） | | 1.0 |  |
| Other provinces | | 3（50.0） | 3（50.0） | | 3.372（0.795,14.294） |  |
| **Nationality** | |  |  | |  | 0.806 |
| Han | | 116(70.7） | 48（29.3） | | 1.0 |  |
| Zhuang | | 69（69.0） | 31（31.0） | | 1.018（0.627,1.652） |  |
| Others/Foreign | | 6（85.7） | 1（14.3） | | 0.515（0.068,3.880） |  |
| **Occupation** | |  |  | |  | 0.127 |
| Famers | | 85（64.9） | 46（35.1） | | 1.0 |  |
| Others | | 62（81.6） | 14（18.4） | | 0.466（0.247,0.879） | 0.018 |
| Unemployed | | 15（71.4） | 6（28.6） | | 0.711（0.265,1.909） |  |
| Retirees | | 29（67.4） | 14（32.6） | | 0.929（0.480,1.797） |  |
| **Pathological type** | |  |  | |  | 0.046 |
| Squamous cell carcinomas | | 181(72.1） | 70（27.9） | | 1.0 |  |
| Adenocarcinoma | | 3（60.0） | 2（40.0） | | 3.433（0.798,14.763） |  |
| Others | | 7（46.7） | 8（53.3） | | 2.268（1.002,5.132） |  |
| **Degree of differentiation** | |  |  | |  | 0.208 |
| Highly | | 161(75.6） | 52（24.4） | | 1.0 |  |
| Moderately | | 16（57.1） | 12（42.9） | | 1.577（0.788,3.155） |  |
| Poorly | | 14（46.7） | 16（53.3） | | 1.639（0.867,3.101） |  |
| **Surgery** | |  |  | |  | 0.029 |
| Yes | | 114(77.0） | 34（23.0） | | 1.0 |  |
| No | | 77（62.6） | 46（37.4） | | 0.590（0.367,0.948） |  |
| **Chronic diseases** | |  |  | |  | 0.487 |
| Yes | | 50（72.5） | 19（27.5） | | 1.0 |  |
| No | | 141(69.8） | 61（30.2） | | 0.822（0.472,1.429） |  |
| **Readmission** | |  |  | |  | 0.007 |
| Yes | | 6（27.3） | 16（72.7） | | 1.0 |  |
| No | | 185(74.3） | 64（25.7） | | 2.340（1.267,4.321） |  |
