# Supplementary material for: Mortality and associated influencing factors among oral cancer patients in western China: A retrospective cohort study from 2016 to 2021
Source: Medicine (Baltimore). 2023 Oct 13;102(41):e35485. doi: 10.1097/MD.0000000000035485 (PMC10578695; doi:10.1097/MD.0000000000035485)
Supplement: Supplementary file 3 [file medi-102-e35485-s003.docx]

Supplemental TABLE 2 Survival rate of 271 oral cancer patients

|  | 2-year survival rate (%) | 5-year survival rate (%) |
| --- | --- | --- |
| **Overall** | 83.8 | 68.5 |
| **Gender** |  |  |
| Male | 81.1 | 71.2 |
| Female | 84.6 | 63.3 |
| **Age** |  |  |
| ≤55 | 86.1 | 78.1 |
| ＞55 | 78.4 | 59.1 |
| **Native place** |  |  |
| Guangxi | 83.0 | 68.9 |
| Other provinces | — | — |
| **Nationality** |  |  |
| Han | 84.1 | 68.5 |
| Zhuang | 78.0 | 66.9 |
| Others/Foreign | — | — |
| **Occupation** |  |  |
| Famers | 77.9 | 61.8 |
| Others | 93.4 | 81.9 |
| Unemployed | 85.7 | 70.3 |
| Retirees | 74.4 | 65.5 |
| **Pathological type** |  |  |
| Squamous cell carcinomas | 82.5 | 70.6 |
| Adenocarcinoma | 60.0 | — |
| Others | 80.0 | — |
| **Degree of differentiation** |  |  |
| Highly | 84.5 | 73.8 |
| Moderately | 71.4 | 50.9 |
| Poorly | 76.7 | 46.5 |
| **Surgery** |  |  |
| Yes | 89.2 | 71.1 |
| No | 74.0 | 63.5 |
| **Chronic diseases** |  |  |
| Yes | 79.7 | 71.9 |
| No | 83.2 | 67.5 |
| **Readmission** |  |  |
| Yes | 68.2 | 20.8 |
| No | 83.5 | 73.3 |
