# Supplementary material for: Mortality and associated influencing factors among oral cancer patients in western China: A retrospective cohort study from 2016 to 2021
Source: Medicine (Baltimore). 2023 Oct 13;102(41):e35485. doi: 10.1097/MD.0000000000035485 (PMC10578695; doi:10.1097/MD.0000000000035485)
Supplement: Supplementary file 4 [file medi-102-e35485-s004.docx]

Supplemental TABLE 4 Stratified analysis of general characteristics for the effects of surgery on survival

| Stratified variable | With surgery vs without surgery | |
| --- | --- | --- |
|  | HR（95%CI） | AHR（95%CI） |
| **Gender** |  |  |
| Male | 0.409(0.223,0.751) | 0.372(0.193,0.720) |
| Female | 1.143(0.537,2.434) | 0.915(0.402,2.081) |
| **Age** |  |  |
| ≤55 | 0.278(0.118,0.655) | 0.240(0.089,0.648) |
| ＞55 | 0.910(0.526,1.577) | 0.842(0.466,1.521) |
| **Native place** |  |  |
| Guangxi | 0.703(0.062,7.919) | - |
| Other provinces | 0.598(0.379,0.944) | 0.583(0.363,0.939) |
| **Nationality** |  |  |
| Han | 0.592(0.334,1.050) | 0.556(0.303,1.020) |
| Zhuang | 0.652(0.318,1.338) | 0.758(0.327,1.759) |
| Others | - | - |
| **Occupation** |  |  |
| Farmers | 0.464(0.252,0.855) | 0.374(0.181,0.776) |
| Others | 1.015(0.352,2.928) | 0.926(0.252,3.397) |
| Unemployed | 0.165(0.019,1.425) | 0.221(0.022,2.180) |
| Retirees | 1.392(0.435,4. 459) | 0.993(0.249,3.966) |
| **Pathological type** |  |  |
| Squamous cell carcinomas | 0.503(0.307,0.822) | 0.550(0.333,0.907) |
| Adenocarcinoma | - | - |
| Others | 2.756(0.314,24.175) | - |
| **Differentiation** |  |  |
| Highly | 0.767(0.443,1.328) | 0.570(0.319,1.017) |
| Moderately | 0.614(0.181,2.082) | 0.951(0.226,3.997) |
| Poorly | 0.319(0.110,0.924) | 0.129(0.026,0.649) |
| **Chronic diseases** |  |  |
| No | 0.538(0.319,0.905) | 0.460(0.261,0.811) |
| Yes | 1.037(0.402,2.678) | 1.142(0.389,3.353) |
| **Readmission** |  |  |
| No | 0.522(0.315,0.865) | 0.476(0.278,0.817) |
| Yes | 1.262(0.464,3.427) | 1.256(0.268,5.897) |
